# A Suggestion for the Treatment of Graves's Disease

**Published:** 1898-06

**Authors:** W. Macphun Semple


### A SUGGESTION FOR THE TREATMENT OF GRAVES'S DISEASE.

BY

W. MACPHUN SEMPLE, M.B., C.M. Glasg.

On the 12th of October last year I was asked to take charge of a case of well marked Graves's disease of some considerable duration, having been under treatment more or less during about eighteen months. There was a history of improvement up to a point. The symptoms were well marked, and I may be allowed to describe them in some detail, as the case appeared to me to be fairly typical. The patient, who is rather short and inclined to be stout, is a woman of considerable mental gifts, who up to the time of her illness had occupied a good position as a lecturer and teacher in one of the best girls' schools in London, and who consequently had a very considerable amount of somewhat trying mental labours to accomplish, to which we may perhaps fairly attribute the onset of the disease; at least, it is a great predisposing cause in these cases.

When I first saw her she was lying on a sofa, and presented the typical features of Graves's disease. The thyroid gland was very much enlarged, the lobe on the right side being somewhat the larger of the two; both lobes were soft and pulpy in consistency, feeling spongy under the fingers on slight pressure; the noise of the blood-rush was very loud and blowing at each systole of the ventricle; the eyes had the prominent appearance so well known, as though something were pushing them out from behind. The usual condition when she was not on guard in the matter was for the white of the eye to show freely. The heart was slightly dilated, shown by the displaced apex-beat, but the sounds were normal and well marked, though fairly rapid. The pulse, which was quite regular with an even and full beat, numbered about 98 to the minute—not nearly so much as the tachycardia seen in many cases, but sufficiently marked when taken with the other symptoms. The skin was extremely dry and harsh, and patient complained very much of it. There was a considerable amount of flatulence and general dyspepsia with a history of years, as also long continued chronic constipation. She complained that the "spring" had gone out of her feet, so that she walked flat-footed. The eye was more prominent on the right side than on the left, the right side being that on which the gland-enlargement was most marked, and during treatment the relationship between the eyes and the gland has been kept in a very noticeable manner. The patient was unable to perform the slightest task without increasing the rapidity of the heart-beat, and sustained conversation had the same effect, or indeed any sustained mental effort, such as reading "solid literature," had the same result. And lastly, the skin was of a peculiarly bronze brown colour, somewhat like that seen in Addison's disease. At first I was inclined to attribute the colour to her having spent some time at the seaside, lying on a chair-lounge exposed to the air and sun; but as the colour did not disappear, but varied with the moods of the disease, some days when she was not so well being more marked, at other times when she was feeling better a more natural colour prevailing, I was led to the belief that the pigmentation must in this case be looked upon as part of the general complaint; and it is to be noted that on the administration of thyroid gland substance, the brown colouration became very marked. It seems to me that there may possibly be some more intimate connection between Graves's disease and Addison's disease than has been so far acknowledged; at any rate, it seems fairly ascertained that in both diseases there is frequently affection of the sympathetic nervous system. In Graves's disease the disease seems most marked in the upper cervical ganglion, and in Addison's disease the trouble lies in the large sympathetic ganglia in the upper abdomen, whilst several of the other marked symptoms, such as tachycardia, are common to both diseases, and the degeneration of the thyroid gland in the one may have its analogue in the gland-degeneration which is so well recognised a result in the other. However, the space at my disposal does not permit of my entering more fully into the pathology of the subject; but in treating either of these two diseases it would be well to bear constantly in mind the undoubted fact that in both the sympathetic ganglia are somehow or other very frequently involved, and anything which can be done to obtain a healthier sympathetic action is likely to have an ameliorating effect on the disease. However, the pathology in both is at present somewhat obscure.

Before coming into my care the patient had been under treatment in London, and seems to have been brought under the influence of the drugs most used in such cases. Thymus gland had been tried without any marked success. Belladonna, whilst relieving some of the symptoms, seemed to increase the exophthalmos. Electricity had been applied to the eyes by means of a double rheophore. Strophanthus and digitalis had been given, and valerian had been tried, combined with a certain amount of rest in the recumbent position. The result of all previous treatment seems to have been to get the patient into a certain improved condition, beyond which further progress seemed somewhat problematical. On taking charge of the case I was given a fair statement of previous treatment, and the result as it appeared to the patient and her friends, and so, to a certain extent, the ground was cleared for me. For some time previous I had been endeavouring to employ Schott baths, combined with what is known as Weir Mitchell treatment, in certain cases, and the result of the combination and modification which I had employed seemed to me to justify my treating this case of Graves's disease by that method, at the same time endeavouring to obtain what possible advantage I might from the use of drugs. Before however beginning this new treatment I tried the effect of small doses of thyroid gland substance, but the result arising from an increased dose was decidedly alarming. Every symptom previously mentioned was aggravated. Pigmentation increased, tachycardia became embarrassing, and the digestive organs were thrown completely out of order. In fact all the symptoms of a very severe case of Graves's disease became apparent, so that I began my treatment as it were *ab initio*.

The patient was kept in the recumbent position, and although she had never been able to digest milk previously, I prevailed upon her to try skimmed milk, beginning with a small quantity, about a pint a day; but to my surprise she took it from the commencement, and it was not long before she was able to take two quarts each day, with the greatest possible benefit to herself. For some weeks the symptoms seemed to vary a great deal from day to day, and did not seem to mend much. At this time I was administering strophanthus and valerianate of zinc, but it was not until I had begun the modified form of Nauheim bathing that I felt confident of any marked improvement. As it was thought unadvisable to have the patient removed to the bath-room, I began treatment by having her thoroughly sponged with the bath water (calcium chloride and sodium chloride), and almost from the first she experienced benefit. The bath water was prepared from the formula recommended by the brothers Schott for making a weak bath, and the temperature I advised was 95° Fahrenheit; the area over the heart, around the gland and eyes receiving the chief attention. To relieve the persistent constipation I gave one half tamar indien as required. In about ten days the patient's appetite improved rapidly; she was now able to take two quarts of skimmed milk daily, and in addition she was able to eat a small meal of solid food at the ordinary meal times. The harshness of the skin became less troublesome, and the tachycardia improved. Both the condition of the gland and the state of the eyes showed improvement, and so I felt justified in not only continuing the treatment, but in increasing the strength of the bath water. About this stage of the treatment I added lactate of iron and iodide of potassium to the drugs employed, continuing the use of strophanthus. Under the combined treatment the patient began to improve rapidly. Lying the whole day in bed, she was now able to do some reading or light sewing without inducing any aggravation of the symptoms. I also about this time began the use of electricity, which I continued, but I do not think that any special benefit has been derived from its employment, although I took very considerable pains to have the current applied, always bearing in mind the doctrine of cervical sympathetic mischief. Sometimes I was inclined to believe that the application of one pole to the seventh cervical and the other over the area of cardiac dulness had some beneficial effect, but regarding that I am not at all prepared to dogmatise. Of the various drugs which I have employed for the purpose of affecting the heart's action I am not at all enthusiastic. Digitalis does not seem to me to exert any beneficial effect, if indeed it does not do real harm. Cactina, paraldehyde I have tried and given up. Strophanthus and convallaria majalis I still use, although, as I have said, even with regard to them I am by no means very enthusiastic, but still I believe they are the best we have at present for the purpose.

The use of lactate of iron, combined with rest in bed, the skimmed milk, and the ability to eat largely which comes to those who drink it, seems to me to have a very valuable effect on the nervous constitution of the patient. The whole nervous tone seemed to undergo a change, and the patient's spirits rapidly improved, so that after about two months she began to feel so well that it was thought wise to allow her to get out of bed during a certain portion of the day.

As she was able to get up, I convinced her that she was now able to be put in a bath, a proposal she readily agreed to, being convinced of the benefit she had derived from even a limited sponging. The first Schott bath was made weak—twenty gallons of water, one pound of common salt, and four ounces of chloride of calcium. The temperature was kept at 95° Fahrenheit, and the duration of the bath was at first six minutes. The first bath was a pronounced success, the patient experiencing a fresh feeling of renewed vigour, and she was further pleased to find that the exertion of the bath and the subsequent drying process, which partook more of the nature of shampooing with a rough bath towel until the skin was rosy, had not increased the heart's action to any marked degree; as a matter of fact when I saw her, a quarter of an hour after the bath, the pulse had only gone up four beats to the minute, or from 88 to 92. The baths proving useful, they were continued for four mornings; then I called a halt for two days, and then a new series of baths, gradually increasing the strength. During the period the patient was able to read and sew in her lounge chair, to walk in her room, and converse as she pleased without experiencing the very slightest inconvenience.

The eyes had gone back very much, and only shewed the white prominently at very unguarded moments. The gland substance was becoming firm and the blood-rush less distinct. The pulse went down to about 88, and was not affected by moderate exercise, and all the other symptoms had greatly improved. The springiness of the foot had partly returned, and the bronze discolouration had almost entirely disappeared. From the ordinary plain calcium bath I proceeded to the stronger effervescing bath, formed by adding a quantity of hydrochloric acid and bicarbonate of soda to the ordinary bath, and I feel convinced that in this case the combined treatment

I have described has had a very marked and beneficial effect. The patient is now, although continuing treatment, in a wonderfully improved condition; she has been able to walk in her garden for a considerable time without inconvenience, and has even walked up and down stairs without experiencing any breathlessness or other disagreeable symptom whatever. All the symptoms which were so marked at the beginning have either disappeared or become most decidedly better. The pulse still remains about an average of 88, but the apex-beat of the heart is now in its normal position, which may be due to the reduction of the dilatation of the heart noted at the beginning, or not, according to the theory held as to the action of the Schott baths. The iodide of potassium seems to have had a good effect on the gland. The dose which I started with, three grains, produced symptoms of a cold in the head, so I increased the dose to eight grains, three times daily, when the disagreeable symptoms passed away, the only peculiarity complained of being an intolerable itching of the back, which ceased when the iodide was stopped or the quantity taken was reduced. To conclude then, the good result which I have obtained I believe to be in a great measure due to the combination of the Schott baths with the Weir Mitchell treatment, the drugs used being helps towards the result, but not in my opinion being at all sufficient to have caused it.

